# Immune Checkpoint Inhibitors and Cardiac Toxicity in Patients Treated for Non-Small Lung Cancer: A Review

**DOI:** 10.3390/ijms21197195

**Published:** 2020-09-29

**Authors:** Grzegorz Sławiński, Anna Wrona, Alicja Dąbrowska-Kugacka, Grzegorz Raczak, Ewa Lewicka

**Affiliations:** 1 Department of Cardiology & Electrotherapy, Medical University of Gdańsk, Debinki 7 Street, 80-952 Gdańsk, Poland; gslawinski@gumed.edu.pl (G.S.); alicja.dabrowska-kugacka@gumed.edu.pl (A.D.-K.); gracz@gumed.edu.pl (G.R.); 2 Department of Oncology & Radiotherapy, Medical University of Gdańsk, 80-952 Gdańsk, Poland; wronania@gmail.com

**Keywords:** immune checkpoint inhibitors, lung cancer, non-small-cell lung cancer, cardiotoxicity, myocarditis, pericarditis, programmed cell death protein 1, programmed cell death-ligand 1

## Abstract

Lung cancer is a major cause of cancer-related mortality worldwide, both in men and women. The vast majority of patients are diagnosed with non-small-cell lung cancer (NSCLC, 80–85% of lung cancer cases). Therapeutics named immune checkpoint inhibitors (ICIs) have revolutionized cancer treatment in the last decade. They are monoclonal antibodies, and those directed against PD-1 (programmed cell death protein 1) or PD-L1 (programmed cell death-ligand 1) have been used in the treatment of lung cancer and significantly improved the prognosis of NSCLC patients. However, during treatment with ICIs, immune-related adverse events (irAEs) can occur in any organ and any tissue. At the same time, although cardiac irAEs are relatively rare compared to irAEs in other organs, they have a high mortality rate. The two most common clinical manifestations of immunotherapy-related cardiotoxicity are myocarditis and pericarditis. Various types of arrhythmias have been reported in patients treated with ICIs, including the occurrence of life-threatening complete atrioventricular block or ventricular tachyarrhythmias. Here, we aim to summarize the incidence, clinical manifestations, underlying mechanisms, diagnosis, and treatment strategies for ICI-associated cardiotoxicity as these issues become very important in view of the increasing use of ICI in the treatment of lung cancer.

## 1. Introduction

Lung cancer is a major cause of cancer-related mortality worldwide, both in men and women. There are basically two main histopathological types of lung cancer, which also differ clinically, i.e., non-small-cell lung cancer (NSCLC, 80–85% of lung cancer cases) and small-cell lung cancer (SCLC, 10–15% of cases). Importantly, more than half of the NSCLC patients have metastases at the time of diagnosis [1].

Immunotherapy has become a milestone in the treatment of lung cancer over the past decade. Ipilimumab (anti CTLA-4) was the first immunotherapy approved by the US Food and Drug Administration (FDA) in 2010 that demonstrated a survival benefit in patients with metastatic melanoma. Cytotoxic T lymphocyte-associated antigen-4 (CTLA-4) is a co-inhibitory molecule presented on stimulated CD4+/CD8+ T-cells competing with CD28 for binding to B7 (CD80/CD86) molecules on the surface of antigen-presenting cells (APCs). *CTLA4* transmits an inhibitory signal to T-cells, whereas CD28 transmits a stimulatory signal. CTLA-4 is involved in attenuating T cell activation and directly facilitates the inhibitory function of regulatory T cells [2,3]. The interaction of CTLA-4 with B7 results in inhibitory signaling, promoting the survival of cancer cells. Inhibition of CTLA-4 restores co-stimulatory signaling through the B7 and CD28 axis. 

Programmed cell death protein 1 (PD-1) is an immune checkpoint receptor expressed on the surface of activated T cells, B cells, and macrophages. PD-1 binds two ligands: programmed cell death-ligand 1 (PD-L1), which is expressed on activated T cells, B cells, dendritic cells, macrophages, and cancer cells, and programmed cell death-ligand 2 (PD-L2), expressed on activated macrophages, dendritic cells and, to a limited extent, cancer cells. The PD-L1 binding to PD-1 causes immunosuppressive effects and allows the tumor to avoid immune destruction by inhibiting the proliferation and survival of cytotoxic T cells and reducing cytokine production (mainly interleukin-2) [4,5]. This mechanism normally prevents autoimmune diseases, but it can also prevent the immune system from destroying cancer cells. The PD-1/PD-L1 pathway plays a crucial role in cancer cells’ immune escape through the PD-1 upregulation. Positive PD-L1 expression in NSCLC patients is observed in 50% to 70% of cases, and high expression in ≥50% of tumor cells in 7.4%–10.6% of NSCLCs patients [6,7,8,9,10,11,12,13,14]. The expression of PD-L1 can be divided into constitutive expression and inducible expression, depending on the intrinsic or extrinsic stimuli. Constitutive expression of PD-L1 in tumor cells is induced by dysregulation of oncogenic or tumor suppressor gene signaling pathways (i.e., through the RAS-MEK signaling pathway), by activation of abnormal transcription factors (i.e., the oncogenic transcription factor MYC), or by genomic aberrations or gene amplifications (i.e., KRAS mutation). Inducible expression refers to the expression of PD-L1-controlled inflammatory signals from tumor cells or other immune cells. These inflammatory factors include interferon gamma (IFN-γ), tumor necrosis factor alfa (TNF-α), and various interleukins (IL-17, IL-27, IL-10, IL-4, IL-2, and IL-10) [15,16,17]. PD-L1 expression also appears to be associated with increased tumor proliferation and aggressiveness, as well as shorter survival times for patients diagnosed with lung adenocarcinoma [18]. 

Therapeutics named immune checkpoint inhibitors (ICIs) are monoclonal antibodies classified into three subgroups, including PD-1 inhibitors (nivolumab, pembrolizumab), PD-L1 inhibitors (durvalumab, atezolizumab, and avelumab), and CTLA-4 inhibitors (ipilimumab). The novel immunotherapy of NSCLC is based on the PD-1/PD-L1 pathway and results in the enhancement of T cell responses and their antitumor activity. Cancer cells are unable to affect activated T cells and the immune response remains active.

ICIs are used to treat various hematological and solid cancers:

- Pembrolizumab—NSCLC, melanoma, head and neck squamous cell carcinoma (HNSCC), classical Hodgkin lymphoma (cHL), primary mediastinal B cell lymphoma (PMBCL), urothelial carcinoma, gastric cancer, esophageal cancer, cervical cancer, endometrial carcinoma, hepatocellular carcinoma (HCC), advanced renal cell carcinoma (RCC), small cell lung cancer (SCLC), Merkel cell carcinoma (MCC), microsatellite instability-high (MSI-H) or mismatch repair-deficient (dMMR) cancers, MSI-H or dMMR metastatic colorectal cancer, tumor mutational burden-high (TMB-H) solid tumors, and cutaneous squamous cell carcinoma (cSCC);
- Nivolumab—NSCLC, SCLC, metastatic melanoma, RCC, cHL, HNSCC, urothelial carcinoma, MSI-H or dMMR metastatic colorectal cancer, and HCC;
- Atezolizumab—NSCLC, SCLC, metastatic urothelial carcinoma, and triple-negative breast cancer (TNBC);
- Durvalumab—NSCLC, SCLC, and metastatic urothelial carcinoma; and
- Ipilimumab—NSCLC, metastatic melanoma, metastatic RCC, MSI-H or dMMR metastatic colorectal cancer, and HCC [19].

The introduction of ICIs has deeply changed the management of lung cancer and significantly improved clinical outcomes and the survival rate in patients with metastatic NSCLC without activating mutations as molecular drivers of the disease. In the first-line setting, ICIs alone—for patients with high PD-L1 expression or in combination with chemotherapy or combined immunotherapy (irrespectively of PD-L1)—have demonstrated an overall survival advantage compared to standard platinum-based regimens. In the second line of treatment, nivolumab, pembrolizumab, and atezolizumab, were shown to prolong overall survival compared to docetaxel. Nivolumab administered in the next line of treatment to patients with advanced NSCLC resulted in a 13.4% overall survival in a 5-year follow-up [20], while pembrolizumab used in such cases resulted in a 15.5% overall survival and 29.6% in previously untreated patients showing high PD-L1 expression [21]. Durvalumab, introduced as a consolidation therapy in patients with stage III NSCLC, provided a significantly longer progression-free survival in 18 months of follow-up, as well as a benefit in overall survival in the mature survival data analysis [22].

Indications for specific ICIs in the treatment for NSCLC are presented in Table 1 (according to the Food and Drug Administration and National Comprehensive Cancer Network recommendations) [23].

## 2. Cardiac Toxicity of ICIs

### 2.1. Pathophysiology

The mechanism of cardiotoxicity associated with ICIs is not yet fully understood. Cardio-immune crosstalk, which may cause myocarditis or heart failure, has been postulated in some preclinical studies [24,25]. Like cancer cells, cardiomyocytes might also employ the PD-1/PD-L1 pathway to prevent hyperactivation of T cells in a physiological state. ICIs, by releasing tumor suppression of T cells, can also ameliorate the same type of suppression by cardiomyocytes, leading to hyperactivation of T cells in the heart. Subsequently, T cell hyperactivation may result in ICI-associated cardiotoxicity. In preclinical models, T cell responses to cardiac antigens (such as desmin or troponin) have been shown to contribute to heart failure through an autoantibody-independent mechanism, with myocarditis and increased myocardial fibrosis [26]. While studies in mice models have shown a deposition of IgG on cardiomyocytes (IgG that recognizes cardiac troponin I), ICI-associated myocarditis in humans appears to be mediated by T cells and macrophages, with no evidence of B cells or antibody-antigen deposits in the cardiac tissue [27]. PD-L1 expression in cardiomyocytes is increased under the influence of cardiac overload and heart disease, including ischemia and left ventricular hypertrophy [28]. In addition, ICIs might also affect pre-existing cardiac disease, for example, ICIs can accelerate or decompensate pre-existing heart failure in susceptible individuals. This may be due to a direct effect on myocardial T cell regulation or to the severity of systemic inflammation, which is frequently documented in patients treated with ICIs.

Although not used in lung cancer, the cardiotoxicity associated with a CTLA-4 inhibitor has also been extensively studied in other studies. Fulminant myocarditis was observed after the CTLA-4 receptor was knocked out in mice [29].

Apart from the infiltration of T cells and macrophages, a change in the expression of multiple chemokine receptors is also observed. Tumor necrosis factor-α, granzyme B, and interferon-γ are overexpressed, which might contribute to cardiac injury [30,31].

### 2.2. Epidemiology

Immune-related adverse events (irAEs) associated with ICIs can occur in any organ and any tissue. Cardiac irAEs are infrequent, but they are characterized by a high mortality rate. Many types of cardiac irAEs have been reported, mainly myocardial injury (predominantly myocarditis), pericardial effusion, arrhythmia, acute coronary syndrome, and systemic vasculitis. In addition, valvular disease, hypertension, conduction disturbances, as well as sudden cardiac death can occur. Initially, the incidence of ICI-related cardiotoxicity was estimated to be less than 1% [32]. Ganatra et al. [33] postulated that the actual incidence of ICI-associated cardiotoxicity is probably underestimated due to nonspecific symptomatology, potential overlap with cardiovascular diseases and other diseases, the challenge of lung cancer diagnosis, as well as a lack of awareness of this condition. Furthermore, in most oncology trials, cardiac parameters have not been prospectively examined. Therefore, the incidence of ICI-associated cardiac complications may be higher in real world practice. An eight-center institutional registry showed that the prevalence of ICI-related myocarditis, which is a complication associated with the worst prognosis, was around 1.14% among all patients undergoing ICI therapy [34]. An analysis of adverse reactions for the six commonly used ICIs revealed that 2.09% were cardiac. Among cardiac adverse events, myocarditis accounted for 14.1% of cases, followed by pericardial complications in 13.6% and conduction abnormalities in 6.86% of cases [19]. According to the VigiBase (the WHO’s pharmacovigilance database), in which 31,321 adverse events were reported in patients treated with all ICI drugs from 2008 to 2018, the most common were myocarditis (0.39%), pericardial disease (0.30%), supraventricular tachycardia (0.71%), and vasculitis (0.26%) [35]. In patients with NSCLC treated with ICI monotherapy in large trials, reported rates for cardiac irAEs were <1% for nivolumab [36], atezolizumab [37], and pembrolizumab [38]. However, cardiac biomarkers and cardiac function were not routinely examined in these studies. Nowadays, in many patients with advanced lung cancer, the combination treatment with chemotherapy and ICIs is often used. Registration trials for pembrolizumab plus chemotherapy in metastatic NSCLC [39] or for squamous NSCLC [40] did not demonstrate increased cardiotoxicity. In the phase 3 study on durvalumab as a consolidation therapy after chemotherapy in patients with stage III NSCLC, cardiac adverse events were reported in 21 patients (4.4%) [22]. However, cardiac parameters were not prospectively evaluated in these studies. Chitturi et al. [41] in a retrospective analysis of 252 patients with various histology lung cancer reported that patients treated with ICIs had a nonsignificant increase (13.3% vs. 10.3%; *p* = 0.66) in the incidence of major adverse cardiac events compared with patients undergoing non-ICI therapy. However, a small sample size and heterogeneous population must be considered when interpreting results from this study. When compared to patients with advanced melanoma, data on cardiac toxicity from ICIs in patients with lung cancer are scarce, and mainly come from case reports. Cohen et al. [42] reported on the rapid progression of fatal myocarditis in a patient with NSCLC that developed 3 weeks after the second administration of pembrolizumab. Zhou et al. [43] collected published case reports and case series of ICI-associated cardiotoxicity, including 14 patients treated with ICIs for lung cancer. Among eight patients treated with nivolumab, two patients developed myocarditis and one was diagnosed with myocardial necrosis; in two patients, acute coronary syndrome occurred, and acute decompensated heart failure occurred in another two patients, while pericarditis complicated by cardiac tamponade was reported in one patient. These events were usually recognized after the first few administrations of nivolumab, but also occurred after cycle 11 or 24. Of the 2 patients treated with pembrolizumab, one patient developed myocarditis and complete atrioventricular block on day 16 after the first administration, and another patient developed pericarditis after the third treatment cycle. One patient developed myocarditis 3 days after the first dose of atezolizumab.

In turn, a retrospective analysis of the VigiBase revealed 613 fatal ICI-associated toxic events, and among patients treated with anti-PD1/PD-L1 (n = 333), most of them had lung cancer: 152 (54%) [44].

## 3. Clinical Manifestation of ICI-Associated Cardiac Toxicity

### 3.1. Myocarditis

The most common clinical manifestation of immunotherapy-related cardiotoxicity is myocarditis, which is associated with poor prognosis, as mortality reaches 27–46% [45,46]. Even worse prognosis was observed in patients undergoing combined ICI therapy (fatality rate up to 76%) [34,47]. Combined ICI therapy is associated with an almost two-fold higher incidence and mortality from myocarditis [35,45,48,49]. Initially, the incidence of ICI-related myocarditis was determined as 0.09%, but later it was estimated at 1–2% [34,49].

Symptoms are non-specific and may include chest pain, fatigue, myalgia, dyspnea due to heart failure and pulmonary edema, palpitations, pre-syncope, syncope, dizziness, hypotension due to cardiogenic shock, altered mental status, and even sudden cardiac death [50]. Subclinical myocarditis has also been described [51].

ICI-associated myocarditis is the most lethal condition from all clinical manifestations of ICI-related adverse effects [44]. In addition, it has significantly worse outcomes than myocarditis in the general population. In the study by Zhang [52], which included 103 patients with ICI-associated myocarditis, 40% of patients developed major cardiovascular adverse events and 16.5% had a cardiovascular death over a follow-up time of 5 months. This data is remarkable compared to the 4.7 years of follow-up in 670 patients with myocarditis of other etiologies, where 15% of patients experienced serious cardiovascular adverse events and 4% of patients died [53]. Approximately 80% of ICI-related myocarditis cases occur within the first 3 months of ICI therapy. In the study by Johnson et al. [49], myocarditis was diagnosed after a median of 17 days (range 13–64 days) from the initiation of immunotherapy. Mahmood et al. reported a median time of myocarditis onset of 34 days (inter-quartile range: 21–75 days) [34]. Moslehi et al. showed a similar median onset (27 days) but with a wider range (5–155 days), with most cases (76%) occurring within the first 6 weeks after treatment initiation [45].

Some conditions and factors may increase the risk of ICI-associated cardiotoxicity, such as combination or sequential immunotherapies, other irAE (myositis, hepatitis), prior exposure to anthracyclines or chest radiotherapy, and the combination of ICIs and anti-VEGF therapies. Additionally, patients with subclinical autoimmunity may be prone to myocarditis, and Khan et al. reported that approximately 14% of patients with lung cancer have a concurrent diagnosis of autoimmune disease [54]. Interestingly, patients after influenza vaccination showed lower rates of ICI-associated myocarditis and had better outcomes [55]. Men are more likely to derive benefit from ICI therapy than women; however, they are also more commonly affected by irAEs than women [30,35,56]. Likewise, the presence of cardiovascular risk factors is postulated as a possible risk factor for ICI-related cardiotoxicity. Diabetes mellitus, obesity, pre-existent cardiac pathology or peripheral arterial disease, history of smoking, and dyslipidemia are the most often mentioned conditions that may be important; however, large sample studies are needed for confirmation [34,50,51,57,58,59,60,61,62,63,64]. In a retrospective study among 196 patients treated with ICIs for lung cancer (mainly with nivolumab), 11% developed major cardiac adverse events following ICI therapy, defined as myocarditis, non-ST-segment elevation myocardial infarction (NSTEMI), pericardial disorders, or new-onset supraventricular tachycardia [65]. A history of coronary disease significantly increased the risk of occurrence of these cardiac adverse events: estimated hazard ratio (HR) 2.79, 95% confidence interval (CI): 1.18 to 6.59; *p* = 0.019. On the other hand, experimental studies indicate a protective role of PD-L1, PD-1, and CTLA-4 against the development of atherosclerotic lesions [66,67]. Thus, the use of ICIs may be associated with an enhanced pro-atherosclerotic inflammatory response, which may result in ICI-related cardiotoxicity in patients with underlying coronary heart disease or in those with cardiovascular risk factors. Patients with lung cancer represent such a group due to the high prevalence of conventional cardiovascular risk factors. Another important factor influencing cardiovascular risk is the use of lung cancer treatments other than immune-based cancer therapy, such as potentially cardiotoxic chemotherapy or radiation therapy. It has been suggested that chest irradiation prior to ICI therapy favors an endogenous antigen-specific immune response [68] and thus may make lung cancer patients more prone to developing cardiac irAEs.

ICI-associated myocarditis can be categorized as fulminant, clinically significant, or subclinical. Fulminant myocarditis is characterized by hemodynamic and/or electrical instability; subclinical myocarditis refers to the disease that was not recognized or treated, with no evidence of clinical consequences [69]. The severity of myocarditis can be classified into four grades (G1–G4). G1 is defined as subclinical myocarditis, and is diagnosed based on abnormal tests results. Symptomatic but clinically stable cases with abnormal tests results can be classified as G2 and G3, while decompensated cases are classified as G4 [70].

One of the typical features of ICI-associated myocarditis is an increase in serum cardiac biomarkers, such as brain natriuretic peptide (BNP) or N-terminal proBNP (NT-proBNP), troponin, and creatine kinase-myocardial band (CK-MB). In the study by Mahmood et al. [34], NT-proBNP was elevated in 66% of patients with diagnosed ICI-associated myocarditis, while Escudier et al. [46] showed an elevated serum level of BNP/NT-proBNP in 100% of patients with ICI-related cardiotoxicity. An abnormal troponin level was observed in 94% of patients with ICI-associated myocarditis [34].

Electrocardiogram (ECG) abnormalities are frequently recorded; however, a normal ECG does not exclude ICI-associated myocarditis. ECG abnormalities were reported in 89% of patients diagnosed with ICI-associated myocarditis. ECG findings include Q wave formation, ST-T changes, R wave decrease, QRS widening, and possible various arrhythmias (sinus tachycardia, supraventricular and ventricular premature beats, atrial fibrillation, ventricular tachycardia, and fibrillation) or conduction disturbances (from bundle-branch block to complete atrioventricular block) [49,71]. Atallah-Yunes et al. [72] reported complete atrioventricular block as the most common complication of ICI-related myocarditis.

Inconsistent echocardiographic findings are reported. Nevertheless, normal echocardiography does not rule out ICI-associated myocarditis. According to Mahmood et al. [34], 51% of patients with ICI-associated myocarditis retained a normal left ventricular ejection fraction (LVEF). In another case series, left ventricular (LV) systolic dysfunction was reported in 79% of patients, the median LVEF was 35%, and half of the patients showed severe LV systolic dysfunction (LVEF < 35%) [73].

Cardiac magnetic resonance imaging (cMRI) is the most useful non-invasive diagnostic modality for myocarditis. It shows myocardial edema and late gadolinium enhancement (LGE) indicating necrosis and scar formation but with a non-ischemic pattern, segmental or diffuse LV contraction abnormalities, and decreased LVEF. Findings consistent with ICI-related myocarditis include:

- High signal intensity on T2-weighted images reflecting edema;
- Myocardium showing greater contrast enhancement than skeletal muscle reflecting hyperemia; and
- LGE reflecting scar.

However, an absence of positive findings on cMRI does not exclude ICI-associated myocarditis. Myocardial fibrosis and scarring, as reflected by LGE, are subacute or chronic sequelae of myocardial inflammation in myocarditis, and some time is required before they are detected on cMRI. LGE is present in less than 50% of patients with ICI-related myocarditis, in comparison to over 80% of patients suffering from non-ICI myocarditis. Interestingly, the sensitivity of ICI-related myocarditis detection on cMRI scans depends on the duration of signs and symptoms. When the examination was performed during the first 4 days of hospital admission, LGE was present only in 21.6% of cases, compared to 72% when cMRI was performed on day 4 of admission or later. Although a prolongation of the time from the onset of clinical symptoms to cMRI is associated with more frequent detection of LGE, such management is not recommended due to a higher risk of serious adverse events [52,74].

Additionally, cardiac 18F-fluorodeoxyglucose (18F-FDG) position emission tomography (PET), which can show active myocardial inflammatory changes, can be helpful if ICI-associated cardiotoxicity is suspected [49]. When combined with computed tomography (CT), PET/CT is a sensitive method and provides both metabolic information from 18F-FDG and anatomic information from CT in one study. Interestingly, a case report published by Arponen et al. showed that if there remains a suspicion of ICI-associated myocarditis after a negative cMRI, PET-CT imaging may be beneficial in confirming the diagnosis. PET-CT may be an alternative imaging modality to cMRI when MRI is contraindicated or not available [75].

The gold standard for the diagnosis of myocarditis remains endomyocardial biopsy (EMB). In patients with ICI-associated myocarditis, it reveals inflammation with interstitial fibrosis and lymphocyte infiltration. T cell infiltrations can be found in the myocardium, cardiac conduction system, interventricular septum, and pericardium [34]. Immunohistochemistry reveals CD3+/CD8+ cells, with a few CD4+ cells, in addition to CD68+ cells representing histiocytes (macrophages). Less frequently, eosinophils are seen, as compared to myocarditis induced by drugs [33,76,77]. EMB is characterized by some technical limitations. Sampling the right ventricular septal wall may miss inflammatory infiltrations, especially in cases of patchy or focal myocarditis associated with ICI. It is suggested that in patients with negative EMB, it should be reattempted if the symptoms of heart failure worsen. EMB is an invasive procedure and carries the risk of a rare but serious complication of cardiac perforation, with rates < 1% in experienced centers. Coronary angiography is often performed concurrently with EMB to rule out significant coronary artery disease. Because of the lower correlation for cMRI and EMB (in 35% of cases) than reported for myocarditis in the broad population, currently, both cMRI and EMB should be considered in the diagnosis of ICI-related myocarditis [78].

Myocarditis can be diagnosed using the following methods, listed here in order of decreasing superiority:

- Tissue pathology in EMB or autopsy (gold standard);
- cMRI showing myocardial oedema and the presence of LGE;
- Echocardiography showing new LV wall motion abnormalities (WMA); and
- Elevated biomarkers.

Positive findings must be supported by laboratory findings, physical examination, and the clinical context. The definitive diagnosis of ICI-associated myocarditis should include at least one of the following:

- Sufficient histopathological evidence in EMB;
- Typical features in cMRI and clinical symptoms accompanied by cardiac biomarkers or ECG; and
- New-onset ventricular WMA in echocardiography, clinical symptoms, elevated cardiac biomarkers, abnormal ECG, and current negative coronary angiography [79].

Bonaca et al. proposed more standardized definitions and categorized suspected cases into one of three groups: definite myocarditis, probable myocarditis, and possible myocarditis [80]. Categorization is hierarchical with a gradually decreasing level of evidence, from EMB and cMRI as the most recommended to diagnose ICI-related myocarditis, through TTE and PET, and ending with cardiac biomarkers, which have the lowest level of evidence to diagnose only possible myocarditis. Detailed information on the division of myocarditis into definite, probable, or possible is given in Table 2.

### 3.2. Pericarditis

The second most common manifestation of ICI-associated cardiotoxicity is pericarditis, which can manifest as isolated pericardial effusion, cardiac tamponade, or perimyocarditis [35,81,82,83,84,85]. Pericardial toxicities occur at a median of 30 days from ICI initiation (interquartile range 8.5–90 days) and are reported more often in males (60% of cases) [35]. Transthoracic echocardiography is a sensitive tool in the diagnosis of pericardial diseases (e.g., to monitor the amount of effusion and to diagnose cardiac tamponade). Some additional information can be obtained using cMRI and cardiac 18F-FDG PET/CT (i.e., to diagnose perimyocarditis). In patients with perimyocarditis, elevated serum troponin levels were commonly found. The ECG may show typical changes for the presence of pericardial effusion, such as low QRS voltage, diffuse concave-upward ST-segment elevation, and tachycardia. Kolla et al. [86] described two patients with lung cancer and cardiac tamponade, which occurred 7 and 9 weeks after starting nivolumab, and postulated that patients with malignant visceral involvement should be carefully monitored due to the risk of rapidly increasing exudate.

### 3.3. Arrhythmias

Various types of arrhythmias have been reported in patients treated with ICIs, including the occurrence of life-threatening complete atrioventricular block or ventricular tachyarrhythmias. The most common is atrial fibrillation (30% of cases), followed by ventricular tachycardia or ventricular fibrillation (27% of cases) and atrioventricular conduction disorders (17% of cases) [46]. Conduction disorders were associated with increased cardiovascular mortality in patients treated with ICIs (80% vs. 16%) [46]. Interestingly, not all patients had arrhythmia as a result of concomitant myocarditis. In these cases, the pathophysiology and mechanism of the arrhythmia remain unclear, and several hypotheses have been made:

- Triggered activity or re-entry mechanism due to fibrosis related to myocardial inflammation;
- Inflammation involving the cardiac conduction system or direct interactions between T cells and cardiac Purkinje cells;
- Systemic inflammation leading to ventricular and supraventricular arrhythmias but without concomitant myocarditis;
- Non-inflammatory LV dysfunction resulting in arrhythmias, especially in patients treated with ICI and conventional chemotherapy;
- Local inflammation caused by myocardial NSCLC metastases; and
- Other cause, i.e., QT interval prolongation, electrolyte imbalance [87].

Once ECG shows PR interval prolongation, bundle branch block, or second-degree atrioventricular block, the threshold for introducing cardiac pacing should be low because conduction disturbances may rapidly progress to advanced heart block. To avoid fatal adverse events, all patients receiving ICIs should undergo regular ECG screening [80].

### 3.4. Other Manifestations

Other clinical manifestations of ICI-associated cardiotoxicity include hypertension, sinus tachycardia, Takotsubo-like syndrome, myocardial ischemia and myocardial infarction, and valvular dysfunction [88,89,90,91]. A rare manifestation of ICI-associated cardiotoxicity can be a coronary spasm, reported in a patient treated with nivolumab [92]. It should be emphasized that some of the cardiac complications listed above are secondary results of ICI therapy, which are rather the consequences of the presented myocarditis/pericarditis.

## 4. Diagnosis of ICI-Associated Cardiotoxicity

Laboratory tests (including cardiac troponin and natriuretic peptides) and repetitive ECGs are suggested for the early diagnosis of subclinical cardiotoxicity associated with ICI, combined with prompt access to TTE in doubtful cases [43,71]. At each visit during ICI treatment, the patient should be inquired about cardiac symptoms and a careful physical examination should be performed.

### 4.1. Electrocardiogram

ECG is characterized with high sensitivity and low specificity, e.g., cardiac arrhythmias are quite common on ECG in the cancer population and do not always indicate cardiotoxicity associated with oncological treatment. ECG abnormalities that may raise suspicion of cardiotoxicity include newly emerging PR interval prolongation, atrioventricular block, supraventricular and ventricular arrhythmias, ST-segment depression, diffuse T-wave inversions, ST-segment elevations mimicking myocardial infarction with ST-segment elevation (STEMI), and new Q waves. Nonspecific T wave changes are the most common ECG abnormalities seen in myocarditis [19].

### 4.2. Imaging Techniques

TTE can reveal new global or segmental WMA, as well as LV systolic and diastolic dysfunction, geometric LV changes in patients with Takotsubo-like syndrome, and pericardial effusion. LV systolic function should be assessed not only by measuring LVEF but also by assessing global longitudinal strain (GLS). Awadalla et al. showed that GLS was reduced in patients with ICI-associated myocarditis who presented both preserved and reduced LVEF [56]. A lower absolute GLS value is strongly associated with higher subsequent risk of major adverse cardiac events (MACEs) among all myocarditis cases [56].

cMRI is the second (after TTE) most often used imaging modality to detect ICI-associated myocarditis, indicating the presence of edema, necrosis, and scar formation.

### 4.3. Laboratory Tests

However, no protocols have been definitively established and there are currently no standard guidelines for active screening for the prevention of ICI-associated cardiotoxicity. Wang et al. [48] proposed cardiac troponin (cTn) measurements before treatment initiation and then after two weeks, 1 month, and 3 months. Puzanov et al. recommended cTn measurements at baseline and weekly for 6 weeks [32]. Gou et al. [71] suggested that noninvasive tests, including cardiac troponin I (cTnI), BNP/NT-proBNP, and ECG, should be performed every 1–2 weeks in the first six weeks of ICI treatment. As cardiac troponin T (cTnT) and creatine kinase (CPK) levels can also be increased in patients with myositis, cTnI is preferred to identify cardiac injury [93]. However, according to the guidelines from the American Society of Clinical Oncology, serial cardiac biomarker testing is not recommended in patients undergoing ICI treatment [70]. A pre-treatment electrocardiogram is recommended but monitoring of troponin levels should be considered in patients initiating combination immune therapies that significantly increase the risk of cardiac toxicity. Additional examinations, such as echocardiography, natriuretic peptides’ level examination, and stress tests, should be performed when indicated by symptoms and signs. Routine testing of troponins and natriuretic peptides in patients undergoing ICI therapy is not supported by current data. However, major efforts are now required to determine which patients are susceptible and most at risk of developing ICI-related cardiotoxicity and on what basis to define the risk groups. It is important at the very beginning, before administering the ICIs, to review the patient history of cardiac diseases and poorly controlled cardiovascular risk factors as well as assess the presence of clinical or subclinical autoimmune disease. Previous chest radiation therapy prior to or concurrent with systemic therapy should also be considered. Patients with NSCLC are often elderly, so cardiovascular disease is to be expected [43,71]. 

## 5. Management of ICI-Associated Cardiotoxicity

Recommendations for the management of ICI-related cardiotoxicity have been independently published by specific scientific societies:

- The American Society of Clinical Oncology (ASCO; 2018) [70];
- The Society for Immunotherapy of Cancer (SITC) Toxicity Management Working Group (2017) [32];
- European Society for Medical Oncology (ESMO; 2017) [94]; and
- National Comprehensive Cancer Network (NCCN) guidelines (2019) [95].

The summary of these documents is presented in Table 3.

General recommendations include:

- Immediate ICI discontinuation in case of demonstrated cardiotoxicity: According to ASCO guidelines considering the potential possibility of progression, and the high mortality rate of ICI-related cardiotoxicity, it would be reasonable to withhold ICIs for all grades of toxicity;
- High-dose corticosteroids (1–2 mg/kg of prednisone) for most patients and intravenous methylprednisone for severe cases should be promptly initiated;
- The patient should be admitted to the hospital and consulted by a cardiologist;
- Guideline-directed management of cardiac symptoms should be provided;
- Patients with elevated troponin or atrioventricular conduction disturbances may require immediate transfer to a coronary care unit; and
- Re-challenging ICIs may be considered in grades 1 and 2 after normalization of the abnormal tests.

The principal strategy to treat ICI-associated cardiotoxicity is to suppress the hyperactive T cell response. For this purpose, corticosteroid therapy is now the first line of immunosuppressive treatment [32,43,70,71]. Depending on the hemodynamic status, in most patients, oral or intravenous prednisone (1–2 mg/kg) is administered and intravenous methylprednisolone (0.5–1.0 g) is recommended for patients with life-threatening conditions (fulminant myocarditis, ventricular tachyarrhythmias, pericarditis with cardiac tamponade). After 3–5 days, it is recommended to gradually reduce the dose of steroids, tapering them over 1 month, and in severe cases, even longer.

However, some patients do not respond well to corticosteroids, and other immunosuppressive regimens should be considered. Until now, immunoglobulin, plasmapheresis, mycophenolate mofetil, tacrolimus, and infliximab have been used as adjunctive therapy in these cases. Infliximab, a chimeric monoclonal antibody that binds to TNF-α, has been used to treat patients with steroid-refractory myocarditis and has shown clinical recovery [96]. However, it is contraindicated in patients with moderate to severe heart failure, as it may result in its deterioration. Since ICI-associated myocarditis and heart transplant rejection show histological similarity, anti-transplant rejection agents, e.g., anti-thymocyte globulin (ATG), have also been used. Agrawal et al. [96] presented two patients with ICI-related myocarditis and unsuccessful steroid therapy in whom ATG allowed remission of cardiogenic shock and malignant arrhythmias. There are also reports of successful use of alemtuzumab in such cases [97]: A monoclonal antibody that targets an antigen known as CD52, a protein antigen found on B and T cells, and these C52-bearing lymphocytes are targeted for destruction after its administration. Recently, Salem et al. also demonstrated the efficacy of abatacept, a CTLA4 agonist, in a patient with metastatic lung cancer who developed corticosteroid-refractory myocarditis after the third administration of nivolumab [98].

In addition to immunosuppressive treatment, additional guideline-directed therapy should be administered as needed. It includes the use of beta-blockers and angiotensin-converting enzyme (ACE) inhibitor/angiotensin receptor blocker in patients with heart failure and antiarrhythmic agents (amiodarone) in case of malignant arrhythmias. Patients who develop cardiac tamponade may require pericardiocentesis or pericardial window placement. Temporary or permanent cardiac pacing is recommended in patients with advanced conduction disturbances. In severe heart failure refractory to vasoactive drugs, an intra-aortic balloon pump (IABP) or extracorporeal membrane oxygenation (ECMO) might be considered.

There is no consensus and it is controversial whether ICI therapy should be reintroduced after cardiotoxicity has resolved. It is generally discouraged; however, Escudier et al. reported that immune therapy was administered again after the first episode of cardiotoxicity in four patients without any recurrences [46]. In each case, such a decision should be made on an individual basis, taking into account the cancer status, the current cardiac function, and the severity of cardiotoxicity. The cardio-oncology team should make the final decision.

## 6. Clinical Outcome

Varricchi et al. [30] postulated that cardiac toxicity associated with ICIs is an emerging problem that requires a close collaboration between cardiologists, immunologists, and oncologists. The number of patients exposed to immunotherapy for cancer, including NSCLC, is expected to dramatically increase in the near future.

There is limited data on the survival and prognosis of patients who experienced cardiotoxicity associated with ICIs. Postulated risk factors linked with a worse prognosis include high serum troponin levels, reduced LVEF, conduction disorders, and combination therapy with ICI. The worst prognosis is associated with myocarditis with a fulminant course.

However, it should be emphasized that despite the observed cardiotoxicity, ICIs are a group of drugs that have significantly improved the survival rate of patients with NSCLC. Rahouma et al., in a meta-analysis of randomized clinical trials of anti-PD/PD-L1 immunotherapy, showed that no statistically significant cardiotoxicity was found with anti-PD/PD-L1 treatment compared to chemotherapy. Lung cancer subgroups showed a better response and survival rates in the immunotherapy group compared to the chemotherapy group [99]. Real world studies (multicenter, observational, retrospective) showed a progression-free survival similar to the results of Keynote-024 (pembrolizumab as first-line treatment) and good patient tolerance [100].

## 7. Conclusions

Lung cancer, which in more than 80% of cases is classified as NSCLC, is the leading cause of cancer-related mortality worldwide. ICI therapy has improved the results of treatment of NSCLC and many other cancer types. Unfortunately, more and more patients are experiencing side effects from this treatment. Although cardiovascular adverse effects are relatively rare, they are associated with serious outcomes. Patients with NSCLC often present concomitant cardiac diseases and cardiovascular risk factors (smoking history, hypertension, dyslipidemia, obesity), which may increase the risk of ICI-related cardiotoxicity. In addition, other treatments used in NSCLC may also be relevant, i.e., chemotherapy, radiotherapy. Therefore, there is a growing need to establish the cardiovascular sequelae in patients with lung cancer treated with immunotherapy and to identify patients at risk of ICI-associated cardiotoxicity who would benefit from closer monitoring. Careful observation, including clinical evaluation, serial measurements of cardiac biomarkers, and ECG, as well as echocardiography, and in case of doubt cMRI and PET, should be included in the recommendations for the management of patients receiving ICI therapy. As cancer treatment protocols become more and more complex, it is likely time for cardio-immuno-oncology to be introduced into the routine management of patients undergoing immunotherapy for cancer treatment.

## Figures and Tables

**Table 1:** Indications (with the level of evidence) for specific ICIs in the NSCLC treatment, according to the Food and Drug Administration (FDA) and National Comprehensive Cancer Network (NCCN) recommendations [23].

Immune Checkpoint Inhibitor (ICI)	Target	FDA Approval	NSCLC Indications	NCCN Guideline Category
Nivolumab	PD-1	2015	Second line regardless of the histological subtype in NSCLC in patients who showed progression despite the platinum-based therapy	1
Nivolumab with Ipilimumab	PD-1 CTLA-4	2020	First-line treatment in metastatic NSCLC with tumors that have PD-L1 expression ≥1% with no EGFR or ALK genomic tumor mutations.	2a
			With two cycles of platinum-doublet chemotherapy, as first-line treatment for patients with metastatic or recurrent NSCLC with no EGFR or ALK genomic tumor mutations.	2a
Pembrolizumab	PD-1	2015	Metastatic NSCLC that progressed after platinum-based therapy or, if appropriate, targeted therapy (EGFR/ALK mutation) and positive for PD-L1	1
			First-line treatment in patients with metastatic NSCLC with high PD-L1 expression (≥ 50%) but no EGFR or ALK mutation	1 2B if PDL-1 1–49%
			First-line treatment in combination with pemetrexed and carboplatin for metastatic non-squamous NSCLC without EGFR or ALK mutation, irrespective of PD-L1 expression	1
			First-line treatment in metastatic squamous NSCLC in combination with carboplatin with paclitaxel/nab-paclitaxel regardless of PD-L1 status	1
			First-line monotherapy in patients with stage 3 NSCLC who are not candidates for surgical resection as well as chemoradiation or metastatic NSCLC with PD-L1 expression ≥1% and no EGFR or ALK mutation	1
Durvalumab	PD-L1	2018	Stage III NSCLC patients for surgically unresectable tumors and whose cancer has not progressed after treatment with chemoradiation	1
Atezoluzimab	PD-L1	2018	Metastatic NSCLC patients with disease progression during or following platinum-containing chemotherapy who have progressed on an appropriate FDA-approved targeted therapy	1
			In combination with bevacizumab, paclitaxel and carboplatin for initial treatment of people with metastatic non-squamous NSCLC with no EGFR or ALK	1
			In combination with paclitaxel protein-bound and carboplatin for initial treatment of people with metastatic non-squamous NSCLC with no EGFR or ALK	1
			First-line monotherapy in patients with metastatic NSCLC, high PD-L1 expression and no EGFR or ALK mutation	1

ALK—anaplastic lymphoma kinase, EGFR—epidermal growth factor receptor, CTLA-4—Cytotoxic T lymphocyte-associated antigen-4, NSCLC—non-small-cell lung cancer, PD-1—programmed cell death protein 1; PD-L1—programmed cell death-ligand 1.

**Table 2:** Proposed division of myocarditis into definite, probable, or possible (modified according to Bonaca et al. [80]). The hierarchical definition reflects a gradually decreasing level of evidence.

	Definite Myocarditis	Probable Myocarditis	Possible Myocarditis
based on emb pathology	tissue pathology consistent with myocarditis	not applicable	not applicable
**OR**			
Based on cMRI	Diagnostic cMRI and clinical symptoms with at least one of: - positive biomarkers, - ECG abnormalities.	Diagnostic cMRI without any of the following: - clinical symptoms, - positive biomarkers, - ECG abnormalities.	Suggestive cMRI without: - clinical symptoms, - ECG abnormalities, - positive biomarkers.
		Suggestive cMRI with at least one of: - clinical symptoms, - ECG abnormalities, - positive biomarkers.	
**OR**			
Based on TTE	TTE with new WMA and all of the following: - clinical symptoms, - positive biomarkers, - ECG abnormalities, - negative angiography for CAD.	TTE with new WMA, clinical symptoms with at least one of the following: - ECG abnormalities, - positive biomarkers.	TTE with new WMA with at least one of: - clinical symptoms, - ECG abnormalities.
**OR**			
Based on PET	not applicable	PET imaging showing patchy cardiac 18F-FDG uptake with clinical symptoms	not applicable
**OR**			
Based on biomarkers	not applicable	not applicable	Elevated biomarkers, no alternative diagnosis with at least one of: - clinical symptoms, - ECG abnormalities.

EMG—endomyocardial biopsy, CAD—coronary artery disease, cMRI—cardiac magnetic resonance imaging, ECG—electrocardiography, 18F-FDG—18F-fluorodeoxyglucose, TTE—transthoracic echocardiography, PET—positron emission tomography, WMA—ventricular wall motion abnormalities.

**Table 3:** Management of immune checkpoint inhibitor-associated cardiotoxicity (modified according to Brahmer et al. [70], Puzanov et al. [32], Haanen et al. [91], and Thompson et al. [92]).

Grade	ECG	Biomarkers	Symptoms	Management	ICI withdrawal
1	Abnormal	Abnormal	None	Cardiac disease and cardio-vascular risk factors controlling	Withhold ICIs
2	Abnormal	Abnormal	Mild	Consider high-dose corticosteroids (prednisone 1–2 mg/kg) Cardiac disease and cardio-vascular risk factors controlling	Withhold ICIs
3	Abnormal	BNP > 500 pg/mL, troponin > 99% institutional normal level	Mild	High-dose corticosteroids (prednisone 1–2 mg/kg) Optimally treat identified cardiac conditions	Withhold ICIs and discontinue permanently
4	Abnormal	Abnormal	Moderate to severe and life-threatening conditions	High-dose intravenous corticosteroids methylprednisolone for at least 3-5 days until cardiac function returns to baseline, after that a slow tapering prednisone dose over at least 4–5 weeks In patients who show a poor response to corticosteroids within 24 h, add other immunosuppressive drug (intravenous immunoglobulins, mycophenolate mofetil, infliximab or anti-thymocyte globulin) or consider plasmapheresis. Additional treatment of detected cardiac conditions should be provided	Withhold ICIs and discontinue permanently

BNP—brain natriuretic peptide; ECG—electrocardiogram; ICI—immune checkpoint inhibitor.
